# Decoding the Multi-Component Synergy of Fu Ling Yin Zi for Anti-Oxidative Stress Applications: Formulation Optimization, Molecular Docking, Cell-Based Validation, and 3D-Printed Dysphagia-Friendly Diets

**DOI:** 10.3390/foods15122206

**Published:** 2026-06-18

**Authors:** Cai You, Yining Feng, Chengjun Wu, Ayyoob Ujala, Siddiki Md Robin Hossain, Qin Hu, Tianzhu Guan, Jia Xu

**Affiliations:** 1 Health Science Center, Ningbo University, Ningbo 315211, China; youcaifighting@163.com; 2 School of Food Science and Engineering, Yangzhou University, Yangzhou 225127, China; mx120241326@stu.yzu.edu.cn (Y.F.); 253603123@stu.yzu.edu.cn (C.W.); ujala.ayoub98@stu.yzu.edu.cn (A.U.); mh25217@stu.yzu.edu.cn (S.M.R.H.); qinhu@yzu.edu.cn (Q.H.)

**Keywords:** Fu Ling Yin Zi, network pharmacology, molecular docking, synergistic antioxidant, 3D printing food

## Abstract

Developing functional foods that address both oxidative stress and physiological challenges like dysphagia is a critical frontier in personalized nutrition. This study investigates the multi-component synergy of Fu Ling Yin Zi (FLYZ), a traditional dietary therapy, and translates its functional properties into a 3D-printed dysphagia-friendly food. Using response surface methodology, the optimal FLYZ formulation was established at a 5:1:5 ratio of *Poria cocos (Schw.) Wolf.*, *Amygdalus communis Vas*, and *Citrus reticulata*. Network pharmacology and molecular docking suggested that FLYZ’s active compounds (e.g., nobiletin, stigmasterol, tangeretin, l-SPD, glabridin, estrone) may mitigate oxidative stress via multiple targets (PTGS2, AKT1, TNF, ESR1, MMP9, and MAOA), with pathway analysis pointing to a potential role of the AKT1/GSK3β/HIF-1α axis. Subsequent in vitro cellular assays demonstrated that FLYZ enhanced antioxidant enzyme activities, reduced intracellular ROS, and modulated the expression of associated genes, supporting a potential link to this pathway. To actualize these functional benefits for patients with swallowing difficulties, a novel 3D-printing ink incorporating FLYZ and walnut oil within a hydrogel matrix (3% xanthan gum, 3% pectin, 1.5% carrageenan) was developed. The printed constructs exhibited excellent shape fidelity and, based on standardized IDDSI fork and spoon tests, were categorized as level 4 (pureed/extremely thick). Furthermore, a simulated in vitro digestion model showed that the colloidal network significantly protected FLYZ’s polyphenols and flavonoids, markedly improving their bioaccessibility and post-digestion antioxidant capacity. Collectively, this work establishes an integrated approach that combines predictive molecular profiling with advanced 3D food printing, thereby supporting the development of future foods tailored for personalized nutrition.

## 1. Introduction

Oxidative stress refers to a pathological state where the dynamic equilibrium between the oxidative and antioxidant systems within the body is disrupted due to alterations in internal and external conditions, leading to excessive production of reactive oxygen species (ROS). Oxidative stress exerts a critical influence on the development and progression of various diseases, including atherosclerosis, hypertension, diabetes mellitus, and cancer. Specifically, oxidative stress induces the activation of cell proliferation and migration signaling pathways and promotes macrophage polarization, facilitating the formation of atherosclerotic plaques [1]. The cardiovascular remodeling induced by oxidative stress can accelerate the progression of hypertension [2]. Additionally, vascular damage and endothelial dysfunction caused by oxidative stress lead to diabetic vascular complications, such as retinopathy, nephropathy, and cardiovascular diseases [3]. Oxidative stress-induced DNA damage and mutations result in uncontrolled cell proliferation, suppressed cell differentiation, and cell death evasion, thereby creating favorable conditions for tumorigenesis [4]. Although there are pharmacological interventions (e.g., vitamin C, vitamin E, and β-carotene), their efficacy is inconsistent and may even lead to potential side effects. Therefore, it is of utmost urgency to seek safe and effective adjunctive regulatory strategies for the auxiliary regulation of oxidative stress.

Traditional Chinese medicine (TCM) dietary therapies, which show remarkable efficacy but few side effects, have a long history in the auxiliary regulation of various diseases. Fu Ling Yin Zi (FLYZ), a TCM dietary therapy for oxidative stress, originates from “Gu Jin Lu Yan” quoted in Volume 20 of “Waitai Miyao”. FLYZ consists of three herbs: *Amygdalus communis Vas*, *Citrus reticulata*, and *Poria cocos (Schw.) Wolf.*, which possess multiple activities, such as antioxidant and anti-inflammatory effects. For example, *Amygdalus Communis Vas* can remarkably enhance the antioxidant defense system and mitigate oxidative stress in adults, as evidenced by its significant effects on improving malondialdehyde levels [5]. *Citrus Reticulata* may diminish oxidative stress-triggered hepatotoxicity via epigenetic regulation of the nuclear factor erythroid 2-related factor 2 (Nrf2)-mediated cellular defense system, and shows crucial potential in combating oxidative stress and related pathological conditions [6]. *Poria cocos (Schw.) Wolf.* potentially suppresses formaldehyde-induced oxidative stress and DNA damage in a dose-dependent way [7]. However, the precise anti-oxidative stress mechanisms of FLYZ remain unclear, and the effective constituents and key targets remain to be identified.

A critical challenge arises in delivering such functional dietary therapies to populations most vulnerable to oxidative stress, such as the elderly and patients with chronic diseases. This demographic frequently suffers from dysphagia (swallowing difficulties), which hinders the safe consumption of conventional foods and oral therapeutics, increasing the risk of malnutrition and aspiration pneumonia [8]. Consequently, there is a significant unmet clinical need for foods that are not only texturally appropriate for safe swallowing but also fortified with bioactive compounds to manage underlying pathological conditions like oxidative stress. Extrusion-based 3D printing has emerged as a versatile food-manufacturing technology perfectly suited to address this dual challenge. It enables the creation of personalized nutrition with appealing visual designs and, most importantly, customizable textures. This approach is particularly valuable for elderly and dysphagic patients, as it allows producers to precisely tailor food consistency and structure to meet specific International Dysphagia Diet Standardization Initiative (IDDSI) texture levels 3–7. Softer, cohesive preparations require little oral processing (levels 3–5), whereas firmer gels and solids demand progressively more chewing and tongue control (levels 6–7) [9]. Critical rheological and textural attributes—including hardness, adhesiveness, cohesiveness, springiness, gumminess, and chewiness—govern safe swallowing, and are efficiently evaluated using IDDSI’s simple, reliable tests, guiding both clinical diet planning and industrial production [10].

This study is therefore built upon the central hypothesis that a 3D-printed, texture-modified food matrix can serve as an effective vehicle for delivering the antioxidant compounds of FLYZ, providing a dual-benefit solution for dysphagic patients who are often at high risk for oxidative stress-related conditions. Since FLYZ is a complex combination of herbs and harbors various multi-target components, conventional approaches are unfeasible for systematically exploring its mechanism against oxidative stress. Therefore, the study will integrate network pharmacology and molecular docking and investigate the underlying anti-oxidative stress molecular mechanisms of FLYZ. To systematically validate this hypothesis, we will first integrate network pharmacology and molecular docking to investigate the underlying anti-oxidative stress molecular mechanisms of FLYZ. Moreover, response surface methodology (RSM) optimization based on synergistic effects will be applied to optimize the FLYZ formulation. Cell-based assays on the mouse embryonic liver cell line (BNL CL.2) will also be carried out using the optimal FLYZ formulation to verify its actual anti-oxidative stress effects and clarify which pathway FLYZ modulates to exert its anti-oxidative stress effects. Building on this foundational evidence, the study will culminate in the development of 3D-printed dysphagia diets based on the optimal FLYZ formulation. These food “inks” will be modified with various colloids (e.g., XG (xanthan gum), PT (pectin), and CG (carrageenan)) and different oils (e.g., camellia oil, flaxseed oil, olive oil, and walnut oil) to achieve ideal printability and textural properties. Finally, comprehensive IDDSI assessments, simulated digestion tests, and bioaccessibility measurements will be conducted to confirm that the resulting gels offer a safe, nutritious, and visually appealing option for individuals with dysphagia, effectively bridging traditional herbal medicine with modern food technology to address a specific clinical need.

In summary, this study demonstrates a systematic methodology that integrates network pharmacology, molecular docking, response surface methodology, cellular validation, and 3D printing technology. It explores the potential of FLYZ-based 3D-printed dysphagia diets as an auxiliary strategy for managing oxidative stress-related conditions. By bridging computational prediction with experimental verification and applied food technology, this work provides a translational model that supports the evidence-based development of traditional Chinese medicine and offers a feasible approach for designing personalized, functionally oriented foods for vulnerable populations.

## 2. Materials and Methods

### 2.1. Materials

*Amygdalus Communis Vas*, *Citrus Reticulata*, *Poria Cocos (Schw.) Wolf.*, XG, PT, CG, camellia oil, flaxseed oil, olive oil, and walnut oil were obtained from a local market in Yangzhou (Yangzhou, China). To ensure standardization, the herbal materials were purchased from the authenticated supplier Beijing Tong Ren Tang in June 2025, with the following batch specifications: *Poria cocos (Schw.) Wolf*. (Batch U265), *Amygdalus communis* (200 g pack), and *Citrus reticulata* (200 g pack). H_2_O_2_ (30%), chloroform, anhydrous ethanol, and isopropanol were purchased from Sinopharm Chemical Reagent Co., Ltd. (Shanghai, China). TRIzol was obtained from Thermo Fisher Scientific (Waltham, MA, USA). A real-time fluorescence quantitative PCR (qRT-PCR) kit and a reverse transcriptional kit were purchased from TransGen Biotech (Beijing, China). Seroxide dismutase (SOD) and catalase (CAT) activity detection kits were bought from Beyotime Biotechnology (Shanghai, China) and Grace Biotechnology (Suzhou, China), respectively. Dimethyl sulfoxide (DMSO), ethylene diamine tetraacetic acid (EDTA), phosphate-buffered solution (PBS) phosphate dry powder, and 3-(4,5-dimethylthiazol-2-yl)-2,5-diphenyltetrazolium bromide (MTT) were made by Beijing Solarbio (Beijing, China). All experiments were carried out in triplicate. This study does not involve ethical review due to not involving humans or animals.

### 2.2. Network Pharmacology Analysis

#### 2.2.1. Exploration and Screening of Potent Components and Associated Targets of FLYZ

FLYZ, a renowned TCM formula, has been widely utilized in clinical regulation. It consists of three herbs, namely Xingren (*Amygdalus Communis Vas*), Chenpi (*Citrus Reticulata*), and Fuling (*Poria Cocos (Schw.) Wolf.*). The TCM Systems Pharmacology (TCMSP) Database was employed to identify the bioactive ingredients and targets within FLYZ. The bioactive constituents of this oral formula were screened per absorption, distribution, metabolism, and excretion (ADME) standards (oral bioavailability (OB) ≥ 30% and drug-likeness (DL) ≥ 0.18) [11]. To make the ingredient collection more exhaustive, references were searched based on related keywords [12,13,14]. Next, the predicted names of targets belonging to the “*Homo sapiens*” species were transformed into a unified format using the Unified Protein Database (https://www.uniprot.org/ (accessed on 14 October 2025)).

#### 2.2.2. Identification and Screening of Key Targets

To identify the common targets associated with both FLYZ and oxidative stress, disease-related targets were first retrieved by searching the keyword “oxidative stress” in multiple databases, including GeneCards (http://www.genecards.org/ (accessed on 16 November 2025)), OMIM (https://omim.org/ (accessed on 30 November 2025)), TTD (http://db.idrblab.net/ttd/ (accessed on 15 October 2025)), PharmGKB (https://www.pharmgkb.org/ (accessed on 3 June 2025)), and BioProject (https://www.ncbi.nlm.nih.gov/bioproject/ (accessed on 5 May 2025)). For GeneCards, a Relevance Score threshold of ≥10 was applied to retain high-confidence oxidative-stress-related targets. For OMIM, TTD, and PharmGKB—which are manually curated and contain relatively fewer entries—all retrieved targets were included without additional filtering. All unique gene entries from oxidative-stress-related projects in BioProject were also included. Targets from all sources were merged, and duplicates were removed to create a consolidated oxidative stress target set. After merging and removing duplicates, the overlapping targets between FLYZ and oxidative stress were determined using Venny 2.1. These common targets were then uploaded to the STRING database (version 11.5) with the organism limited to “*Homo sapiens*” to obtain a protein–protein interaction (PPI) network [15]. The PPI data were imported into Cytoscape 3.9.1 for further analysis. Key clusters within the network were identified using the MCODE plugin with the following criteria: degree cutoff = 2, node score cutoff = 0.2, and k-core = 2, and max depth = 100. Subsequently, hub targets were screened by calculating centrality parameters—including degree, betweenness, and closeness—using the CytoHubba and CentiScaPe 2.2 plugins.

#### 2.2.3. Network Construction and Topological Analysis

A “herb–compound–target” network was constructed in Cytoscape 3.9.1 to visualize the interactions among FLYZ herbs, their bioactive compounds, and the common targets identified in Section 2.2.2. The topological properties of this network, as well as of the PPI network, were systematically evaluated using built-in tools and plugins (NetworkAnalyzer, CentiScaPe 2.2). Key topological parameters—such as degree, betweenness centrality, and closeness centrality—were calculated to characterize node importance and network structure, facilitating the identification of functionally central compounds and targets in the anti-oxidative stress activity of FLYZ.

#### 2.2.4. Gene Ontology (GO) and Kyoto Encyclopedia of Genes and Genomes (KEGG) Pathway Enrichment Analyses

To further understand the functional roles of the common targets in biological pathways, GO and KEGG enrichment analyses were performed on the core anti-oxidative stress targets of FLYZ using DAVID 6.8 (https://david.ncifcrf.gov/ (accessed on 19 February 2022)). These analyses helped to clarify the potential anti-oxidative stress mechanisms by examining biological processes (BP), cellular components (CC), molecular functions (MF), and KEGG pathways. A *p* value < 0.05 was set as the threshold for statistical significance. Pathways meeting this criterion were considered to be meaningfully associated with FLYZ activity. The resulting data were ranked by −log10(*p* value), with a smaller *p* value indicating stronger enrichment and greater biological relevance. The top 10 GO terms and KEGG pathways were selected for further investigation.

#### 2.2.5. Molecular Docking

To explore potential interactions between the active components and key targets, molecular docking was performed using AutoDock Vina (Version 1.5.6) [16]. The six hub targets identified in Section 2.2.4 (PTGS2, AKT1, TNF, ESR1, MMP9, MAOA) and their corresponding active ingredients were selected. The crystal structures of the targets were retrieved from the RCSB Protein Data Bank (https://www.rcsb.org/ (accessed on 17 November 2025); PDB IDs: 5IKR, 1UNQ, 1VYR, 1A52, 6ESM, 2Z5Y, respectively). The native co-crystallized ligands, where available, were extracted and re-docked into their respective binding sites to validate the docking protocol; the root-mean-square deviation (RMSD) between the docked and crystal conformations was less than 2.0 Å, confirming the reliability of the method. Protein structures were prepared using AutoDock Tools by removing water molecules, adding polar hydrogens, and assigning Gasteiger charges. A grid box was set to encompass the known active site of each protein, with dimensions adjusted to allow sufficient conformational sampling. Docking simulations were performed, and the binding energy (ΔG, kcal/mol) for each compound–target pair was calculated. A lower (more negative) binding energy indicates a more favorable predicted interaction. A binding energy ≤−5.0 kcal/mol is commonly considered to suggest the potential for spontaneous binding [17]. The docking poses were visualized and analyzed using PyMOL 2.4.0 and Discovery Studio 2018 to identify potential interaction modes.

### 2.3. Optimization of FLYZ Formulation and Antioxidant Activity Evaluation

#### 2.3.1. Preparation of FLYZ

The three herbs of FLYZ (*Amygdalus Communis Vas*, *Citrus Reticulata*, and *Poria cocos (Schw.) Wolf.*) were dried using an electrically heated constant-temperature air blowing drying oven (DHG-9076A, Jinghong Laboratory Equipment Co., Ltd., Shanghai, China) at 60 °C overnight following Guan et al. [18]. Thereafter, the herbs were ground into powder, sieved through a 60-mesh screen, and then stored at room temperature (RT) for subsequent use. With a fixed solid-to-liquid ratio of 1:10, various proportions of the herbal powder were prepared prior to a heating and ultrasonic process at 300 W and 50 °C for 2 h. Then the extract was centrifuged at 8000 rpm with a high-speed refrigerated centrifuge (Sigma 1-14, Sigma Laborzentrifugen GmbH, Osterode am Harz, Germany) for 10 min to obtain the supernatant.

#### 2.3.2. ABTS^+^ Scavenging Capacity Assay and Analysis of Antioxidant Synergism

The ABTS^+^ scavenging capacity was determined according to the method described by Guan et al. with slight modifications [19]. Briefly, the ABTS^+^ working solution was prepared by reacting 7 mM ABTS^+^ and 2.45 mM potassium persulfate (K_2_S_2_O_8_) at a 1:1 (*v*/*v*) ratio. The mixture was incubated in the dark at room temperature for 12–16 h. Prior to use, the solution was diluted with 95% ethanol until the absorbance at 734 nm reached 1.40 ± 0.02. For the assay, 10 μL of the test sample supernatant was mixed with 200 μL of the diluted ABTS^+^ working solution. After incubating in the dark for 6 min at room temperature, the absorbance was measured at 734 nm using an Infinite F50 microplate reader (Tecan Group Ltd., Männedorf, Switzerland). A control was prepared by replacing the sample with distilled water. All measurements were performed in triplicate. The ABTS^+^ radical scavenging rate was calculated using the following formula:

(1) $${ABTS}^{+}\;radical\;scavenging\;rate\;\left(\%\right)=\left[1-\frac{\left(A_{1}-A_{2}\right)}{A_{0}}\right]\times 100\%$$

where *A*_1_ is the absorbance of the sample mixture, *A*_0_ is the absorbance of the control (water instead of sample), and *A*_2_ is the absorbance of the sample background (water instead of ABTS^+^ working solution).

Based on the ABTS^+^ scavenging rates obtained for different FLYZ formulations, the potential synergistic antioxidant effects among the components were evaluated using the Chou–Talalay combination index (CI) method via the CompuSyn software, version 1.0.1 (ComboSyn, Inc., Ridgewood, NJ, USA) [20]. The combination index (CI) value of <1, =1, and >1 indicates synergy, an additive effect, and antagonism, respectively.

#### 2.3.3. Single-Factor Experiments

Single-factor experiments were performed to identify the optimal formulation of FLYZ. Different amounts of *Poria Cocos (Schw.) Wolf.* (0.05, 0.1, 0.15, 0.2 g), *Amygdalus Communis Vas* (0.05, 0.1, 0.15, 0.2, 0.25 g), and *Citrus Reticulata* (0.05, 0.1, 0.15, 0.2, 0.25 g) were precisely weighed. While keeping other factors at their intermediate levels, each single-factor experiment was carried out separately. Sample preparation followed the procedure described in Section 2.3.1. The supernatant was diluted 10-fold, and the ABTS^+^ scavenging rate was then calculated. All experiments were performed in triplicate to ensure reproducibility.

#### 2.3.4. Response Surface Methodology (RSM) Design

A response surface design was implemented using Design Expert 12 software to evaluate the effects of three factors: *Poria cocos (Schw.) Wolf.* (coded as A, mass range 0.15–0.25 g), *Amygdalus communis Vas* (coded as B, mass range 0.05–0.15 g), and *Citrus reticulata* (coded as C, mass range 0.15–0.25 g), with the ABTS^+^ scavenging rate as the response variable. The total mass of the mixture was fixed at 0.55 g.

#### 2.3.5. Antioxidant Synergism

Based on the Chou–Talalay combined drug theory, the synergistic antioxidant effects of different FLYZ formulations were explored via the CompuSyn program [20]. The combination index (CI) was calculated to quantify the degree of synergy, additivity, or antagonism among the components in the formulations.

#### 2.3.6. Cell Culture and Cytotoxicity Experiment

BNL CL.2 from Dalian Meilun Biotechnology Co., Ltd. (Dalian, China) was cultured in Dulbecco’s modified Eagle’s medium (DMEM)-high glucose (VivaCell, Shanghai, China) containing 10% fetal bovine serum (FBS, VivaCell) and 1% penicillin/streptomycin (P/S, TransGen, Beijing, China) at 37 °C in a 5% CO_2_ and 95% air atmosphere. To evaluate the cytotoxicity of H_2_O_2_ and FLYZ as well as the protective effect of FLYZ against H_2_O_2_-induced cell damage, cell viability was assessed using the MTT method [21]. Briefly, the BNL CL.2 cells were seeded at a density of 1 × 10^4^ cells/well in 96-well culture plates and incubated for 18 h with various treatments. The cells were then exposed to H_2_O_2_ (200, 400, 600, 800, or 1000 μM) or FLYZ (0.5, 1, 2, 4, or 8 mg/mL) for 24 h. To evaluate the protective effect of FLYZ, cells were pre-treated with H_2_O_2_ (600 μM) for 4 h, and co-cultured with FLYZ at varying concentrations for 20 h. The culture medium was substituted with a fresh one containing 5 mg/mL MTT, and then incubation lasted for another 4 h. After the removal of the medium, 100 µL of DMSO was added to each well and mixed completely. Then the absorbance at 540 nm was measured via a Varioskan LUX Multimode Microplate reader (Thermo Fisher Scientific Inc., USA).

#### 2.3.7. Biochemical Analysis of SOD and CAT Activities

The BNL CL.2 cells were pre-treated with H_2_O_2_ or FLYZ at various concentrations. The activities of CAT and SOD were measured using commercial kits (Cat. no. S0101S, G0105W48) according to the manufacturers’ protocols. SOD activity was measured using the xanthine oxidase method, while CAT activity was determined using the colorimetry method.

#### 2.3.8. Determination of Intracellular Reactive Oxygen Species (ROS) Levels

Intracellular ROS levels were quantified using a DCFH-DA probe (Bioswamp, Wuhan, China, 10 μM, 25 min) via a CytoFlex S flow cytometer (Beckman Coulter, Brea, CA, USA), and data were analyzed using FlowJo software, v10.9.0 (Ashland, OR, USA).

#### 2.3.9. RNA Extraction and qRT-PCR

The BNL CL.2 cells were incubated in 6-well plates for 18 h, then pre-treated with 600 μM H_2_O_2_ for 4 h, and exposed to FLYZ (0, 2, 4, or 8 mg/mL) for 20 h. Then, the BNL CL.2 cells were collected. Purified RNA was extracted and accurately quantified using a Nanodrop spectrophotometer. The primers (Sangon Biotech, Shanghai, China) included TGTTTCTACTGTGGGCAGCA and TGGTCG CGTCAGTCCTTAAT (Akt1, Mus); GCTGGAGTACACACCTACCG and GGA GGGATAAGGATGGTGGC (GSK3β, Mus); GGTTCCAGCAGACCCAGTTA and ATGCCTTAGCAGTGGTCGTT (HIF-1α, Mus); CGATGCCCCCATGTTTGTGA and GAGCCCTTCCACAATGCCAA (GAPDH, Mus). qRT-PCR was conducted using SYBR Green as previously described. Three biological replicates were analyzed for each genotype, with GAPDH serving as the normalization reference. The mRNA levels were relatively quantified using the comparative C_T_ method.

### 2.4. Development and Functional Evaluation of 3D-Printed Level 4 Dysphagia Foods

#### 2.4.1. Preparation of Food Ink Formulations

Colloid- and nutrient-based hydrogels formulated for extrusion are referred to as food inks for 3D-printed foods. To optimize texture for dysphagia diets, we performed initial screening of ink formulations by adjusting hydrocolloid levels. PT (1, 2, 3, 4, 5 g/100 g), XG (1, 2, 3, 4, 5 g/100 g), and CG (0.5, 1, 1.5, 2, 2.5 g/100 g) were dispersed in deionized water and stirred magnetically at 60 °C until fully hydrated. Post-heating, inks were cooled to RT, wrapped to prevent evaporation, and stored at 4 °C for 12 h before the assessment of appearance.

#### 2.4.2. Texture Profile Analysis (TPA)

Sample geometry significantly affects the comparability of TPA results. Therefore, cylindrical samples (20 mm diameter × 20 mm height) were prepared according to a previously reported method [22]. Texture analysis was performed using a TMS PRO texture analyzer (Food Technology Corporation, Sterling, VA, USA) equipped with a 20 mm diameter cylindrical probe. The test was conducted at room temperature with the following settings: pre-test speed 2.0 mm/s, test speed 1.0 mm/s, post-test speed 1.0 mm/s, compression strain 60%, and trigger force 0.05 N. Using the standard TPA macro provided by the instrument software, key texture parameters—including hardness, adhesiveness, cohesiveness, springiness, gumminess, and chewiness—were calculated. Each formulation was measured in triplicate.

#### 2.4.3. 3D Printing Procedure and Ink Properties

Inks were printed via a Luckybot One extrusion-based 3D printer (Wiiboox Technology Co., Ltd., Nanjing, China) using a 0.84 mm nozzle. CAD models (butterfly, honeycomb, gecko) were generated in Ultimaker Cura. Printing parameters were: nozzle height 1 mm; layer height 0.55 mm; deposition speed 20 mm/s; extrusion rate 1 mL/min. After a 15 min rest at RT, print fidelity was evaluated with linear infill. Photographs were captured on a food-grade glass plate under uniform LED illumination (144 LED panel).

#### 2.4.4. IDDSI Tests for Dysphagia Texture Classification

IDDSI testing is imperative for classifying dysphagia-appropriate foods, as the IDDSI framework provides the standardized categorization system [23]. This framework defines 8 levels of food textures and drink consistencies tailored for dysphagic individuals. To characterize the 3D-printed ink formulations for oral–pharyngeal processing, three IDDSI-aligned tests were conducted: spoon tilt, fork drip, and fork pressure. All tests were performed at room temperature (22 ± 1 °C), within 30 min after 3D printing to ensure consistency.

Sample preparation and dimensions. For all IDDSI tests, the 3D-printed samples were prepared as cuboids of 15 mm × 15 mm × 15 mm (length × width × height), which aligns with adult choking safety standards (particle size ≤ 15 mm in any dimension). These dimensions were used for the fork pressure test. For the spoon tilt and fork drip tests, approximately 5 mL (spoonful) of the gel sample was taken directly from the printed structure using the test utensil.

Spoon tilt test. A standard metal tablespoon (15 mL capacity, 45 mm bowl length, 32 mm bowl width) was used. The spoon was filled with approximately 5 mL of the gel sample, leveled with a spatula, and then tilted slowly to 90° over a plate. The behavior of the sample was observed: whether it held its shape, slid off intact, left residue, or flowed/dripped. The test was repeated three times per formulation.

Fork drip test. A standard metal dinner fork (four tines, tine length 25 mm, inter-tine gap 4 mm) was used. The fork was inserted vertically into the gel sample to a depth of approximately 10 mm, then lifted and held horizontally for 10 s. The flow of the sample through the tines was observed: whether it remained piled on the fork without dripping, flowed slowly through the tines, or dripped rapidly. Each formulation was tested three times.

Fork pressure test. A standard metal fork (same specifications as above) was used. The 3D-printed cuboid sample (15 mm × 15 mm × 15 mm) was placed on a flat plate. Thumb pressure was applied through the fork tines onto the sample. The ease of mashing and the behavior of the sample under pressure were observed. Specifically, the pressure required to deform the sample was graded as: minimal thumb pressure (no nail blanching), moderate pressure (nail blanching), or firm pressure (significant thumb effort). The samples were also observed for whether they passed through the 4 mm inter-tine gap after compression. Each formulation was tested three times.

Replicates and assessor information. All IDDSI tests were performed in three independent replicates (*n* = 3 for each formulation), with each replicate prepared from a separately printed batch. The evaluations were conducted by two trained assessors (laboratory staff familiar with the IDDSI framework and dysphagia food classification). Discrepancies between assessors were resolved by consensus.

Classification criteria. The IDDSI framework defines level 4 (pureed/extremely thick) foods as: (i) non-pourable, holds shape on a spoon; (ii) slides off the spoon intact without dripping or residue; (iii) does not flow through fork tines (or only minimal flow); (iv) can be mashed with minimal thumb pressure using a fork; and (v) no lumps or liquid separation. Formulations meeting these criteria were classified as Level 4.

#### 2.4.5. Antioxidant Activity of 3D-Printed Gels with Different Oils

The antioxidant activity of the 3D-printed gels, in which the base oil was substituted with camellia oil, flaxseed oil, olive oil, or walnut oil, was evaluated. The ABTS^+^ scavenging capacity was determined as described in Section 2.3.2, using 10-fold diluted gel supernatants. Additionally, the total phenolic content (TPC) and total flavonoid content (TFC) of these gel samples were determined to provide a complementary phytochemical basis for the antioxidant activity.

#### 2.4.6. Determination of TPC and TFC

TPC was quantified in the dark at RT via the Folin–Ciocalteu assay [24]. Briefly, 0.5 mL of each sample and 1 mL of Folin–Ciocalteu reagent (100%) were mixed at RT for 3 min. Next, 5.0 mL of 1 M Na_2_CO_3_ was added, and the final volume was adjusted to 10 mL with deionized water. The mixture was incubated for 1 h, after which absorbance was read at 765 nm against a reagent blank. A series of gallic acid standards (5–200 µg/mL) was used to construct a calibration curve, and results are expressed as mg gallic acid equivalents per g of sample (mg GAE/g).

TFC was measured in the dark at RT using an aluminum nitrate colorimetric assay [25]. A total of 1 mL of each sample, 1 mL of 60% ethanol, and 0.5 mL of 5% NaNO_2_ was added, vortexed, and incubated for 6 min. Next, 0.5 mL 10% Al(NO_3_)_3_ was mixed in and allowed to stand for another 6 min in the dark. Finally, 4.0 mL of 4% NaOH was introduced, vortexed, and left to stand for 15 min. Absorbance was measured at 510 nm using deionized water as the blank. A rutin standard curve (5–100 μg/mL) enabled TFC calculation, reported as mg rutin equivalents per g of sample (mg RE/g).

#### 2.4.7. In Vitro Simulated Gastrointestinal Digestion

An in vitro simulated gastrointestinal digestion model, comprising oral, gastric, and intestinal phases, was employed to investigate the release profiles of bioactive compounds from the 3D-printed gels. The assay was conducted following a previously reported protocol with slight modifications [26]. For the oral phase, each gel sample (control and XG/PT/CG inks containing camellia oil, flaxseed oil, olive oil, or walnut oil, cut into 1 mm × 1 mm pieces) was mixed with simulated salivary fluid (SSF) at a 1:1 (*w*/*w*) ratio. The SSF contained 0.5 M KCl, 0.5 M KH_2_PO_4_, 1 M NaHCO_3_, 0.15 M MgCl_2_(H_2_O)_6_, 0.5 M (NH_4_)_2_CO_3_, 0.3 M CaCl_2_, and mucin (30 g/L). The pH was adjusted to 6.8, and samples were incubated at 37 °C with agitation at 100 rpm for 10 min. The oral bolus was then subjected to the gastric phase by mixing with an equal weight of simulated gastric fluid (SGF). The SGF was composed of 0.5 M KCl, 0.5 M KH_2_PO_4_, 1 M NaHCO_3_, 2 M NaCl, 0.15 M MgCl_2_(H_2_O)_6_, 0.5 M (NH_4_)_2_CO_3_, and 0.3 M CaCl_2_. Pepsin was added to achieve a final activity of 3000 U/mL, and the pH was adjusted to 3.0. The gastric digestion was performed at 37 °C with agitation at 100 rpm for 2 h, after which the reaction was terminated by rapidly raising the pH to 7.0.

For the intestinal phase, the gastric chyme was mixed 1:1 (*w*/*w*) with simulated intestinal fluid (SIF). The SIF contained 0.5 M KCl, 0.5 M KH_2_PO_4_, 1 M NaHCO_3_, 2 M NaCl, 0.15 M MgCl_2_(H_2_O)_6_, 0.3 M CaCl_2_, pancreatin (250 U/mL), and bile salts (5 mg/mL). The pH of the mixture was adjusted to 7.0, followed by incubation at 37 °C with stirring at 100 rpm for 2 h. After each digestion phase, the sample was centrifuged at 8000 rpm for 10 min. The resulting supernatant was collected and stored for subsequent analysis. The bioaccessibility of phenolic compounds after the simulated intestinal digestion was calculated as follows:

(2) $$Bioaccessibility\left(\%\right)=\frac{Final\;TPC\;or\;TFC\;after\;intestinal\;digestion}{initial\;TPC\;or\;TFC\;before\;digestion}\times 100$$

### 2.5. Statistical Analysis

All experiments were conducted in biological triplicate. In multiple comparisons of normally distributed data among more than two groups, one-way analysis of variance (ANOVA) with Tukey’s post hoc test was utilized. The data were presented in the form of mean ± standard deviation (SD) and analyzed in GraphPad Prism 9.5.0 (San Diego, CA, USA). Statistical significance was defined as * *p* < 0.05, ** *p* < 0.01, and *** *p* < 0.001.

## 3. Results and Discussion

### 3.1. Mechanistic Insights from Network Pharmacology Analysis

#### 3.1.1. Screening of Active Compounds and Potential Targets

Using oral bioavailability (OB) ≥ 30% and drug-likeness (DL) ≥ 0.18 as screening criteria, a total of 30 active compounds were identified from FLYZ. These compounds were derived from the constituent herbs as follows: 16 from *Amygdalus communis Vas*, 9 from *Citrus reticulata*, and 6 from *Poria cocos (Schw.) Wolf*. Notably, sitosterol was present in multiple herbs within the formula and was hypothesized to contribute significantly to the anti-oxidative stress activity. The corresponding targets of these active compounds were retrieved and standardized using the UniProt database. After removing duplicates, 103 unique targets associated with FLYZ were obtained for further analysis.

**Figure 1:**
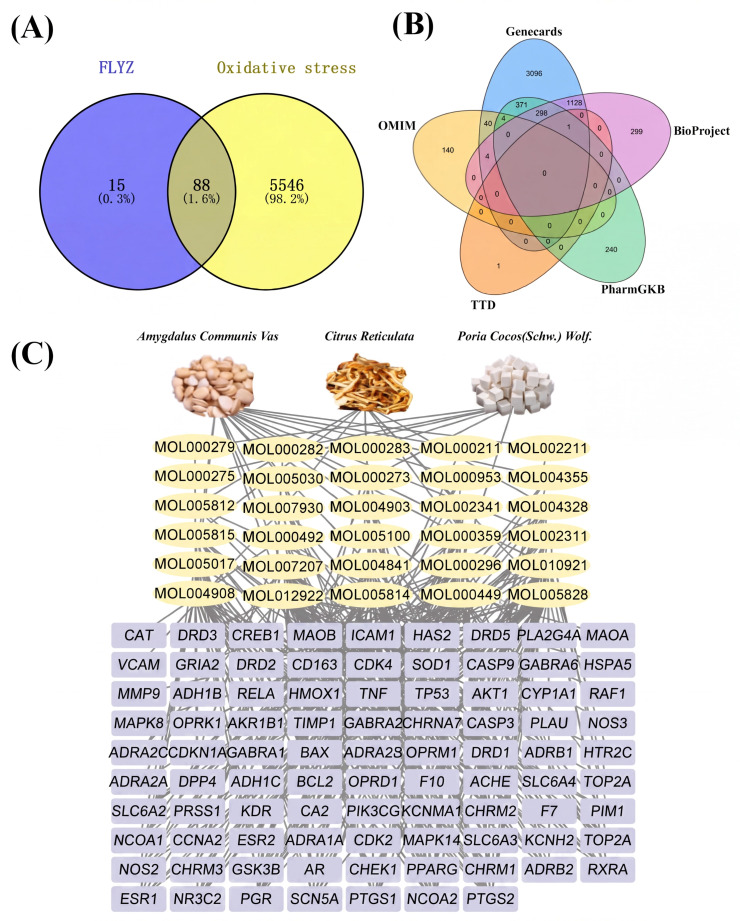
Integrative network pharmacology analysis reveals potential anti-oxidative stress targets and compound-target interactions of FLYZ. (**A**) Venn diagram showing the overlapping targets between FLYZ and oxidative stress. (**B**) Distribution of oxidative stress-related genes collected from multiple databases. (**C**) Herb–compound–target interaction network illustrating the multi-component, multi-target characteristics of FLYZ.

Potential targets related to oxidative stress were collected from the GeneCards, OMIM, TTD, PharmGKB, and BioProject databases using “oxidative stress” as the search term. This yielded 5634 targets, with 4954 from GeneCards, 203 from OMIM, 2 from TTD, 917 from PharmGKB, and 1733 from BioProject (Figure 1B). An intersection analysis between the 103 FLYZ-related targets and the 5634 oxidative stress-related targets identified 88 common targets (Figure 1A), which were considered potential key mediators of FLYZ’s effects and were subjected to subsequent investigation.

#### 3.1.2. Construction and Analysis of the Herb–Compound–Target Network

To elucidate the molecular basis of the anti-oxidative stress effects of FLYZ, an herb–compound–target interaction network was constructed, incorporating three herbs, 30 active compounds, and 88 common targets (Figure 1C). In the network, compounds and targets are represented by yellow ovals and purple rectangles, respectively. The resulting network comprised 121 nodes and 307 edges, reflecting the multi-component, multi-target characteristics of FLYZ. Topological analysis of the network was performed using the NetworkAnalyzer plugin in Cytoscape. Nodes were ranked in descending order based on their degree centrality. Key compounds with the highest connectivity were nobiletin, stigmasterol, tangeretin, l-SPD, glabridin, and estrone, suggesting their potential central roles in the anti-oxidative stress activity of FLYZ (Table S1). These highly ranked components probably play the most significant roles in the anti-oxidative stress process and warrant further investigation and study. For example, nobiletin has been reported to alleviate oxidative stress by suppressing the expression of NADPH oxidase subunits (p67phox, p22phox, and p91phox) [27], while stigmasterol modulates cellular ROS levels and upregulates key antioxidant and survival-related factors (Forkhead box O3a, CAT, and Bcl-2) in neuronal models [28].

#### 3.1.3. PPI Network Analysis and Hub Target Identification

**Figure 2:**
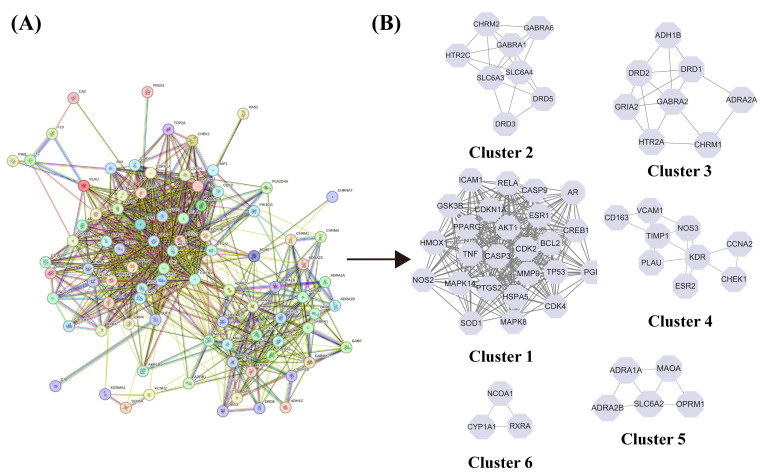
PPI network and functional modules of the common targets. (**A**) PPI network constructed from the 88 overlapping targets between FLYZ and oxidative stress. (**B**) Key functional sub-clusters identified within the PPI network using the MCODE clustering algorithm. Colors in the network are based on STRING’s default classification.

The 88 common targets shared between FLYZ and oxidative stress were used to construct a PPI network using STRING. The resulting network contained 87 nodes and 824 edges, with a highly significant PPI enrichment *p* value (<1.0 × 10^−16^), indicating strong biological connectivity among the targets (Figure 2A). For further functional module analysis, the network was imported into Cytoscape 3.9.1 and analyzed using the MCODE clustering algorithm, which identified six distinct sub-clusters (Figure 2B). To pinpoint the most influential targets within the network, key topological parameters—degree, betweenness centrality, and closeness centrality—were calculated using the CytoHubba and CentiScaPe 2.2 plugins. Based on the combination of these metrics, six targets with the highest connectivity and centrality were selected as hub targets: AKT1, TNF, PTGS2, ESR1, MMP9, and MAOA.

#### 3.1.4. Functional Enrichment Analysis of Potential Targets

**Figure 3:**
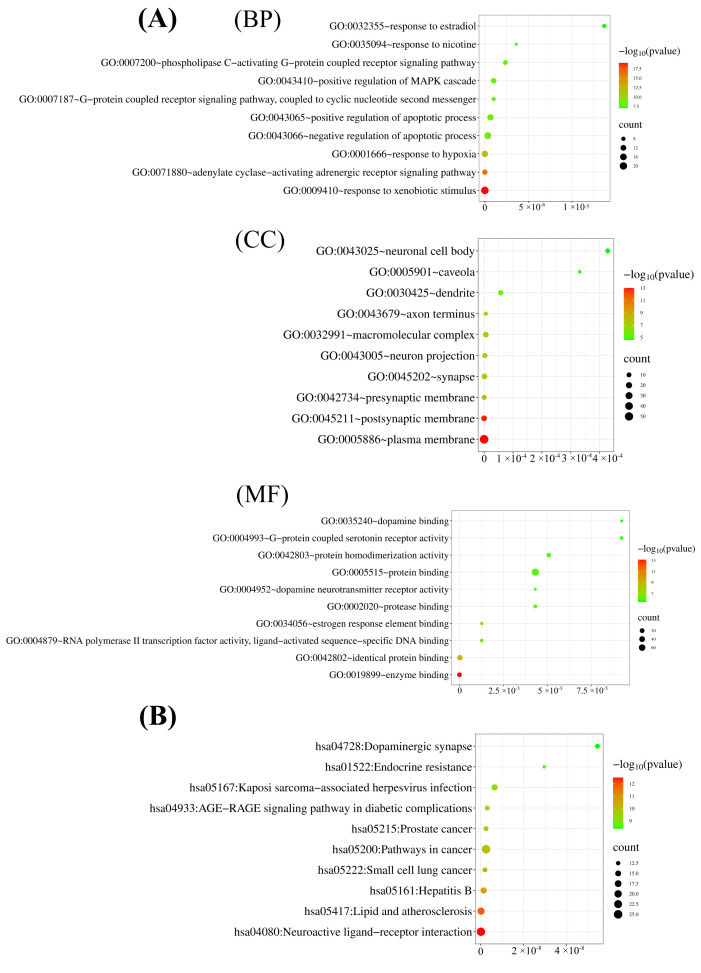
Functional enrichment analysis of the common targets of FLYZ. (**A**) The top 10 significantly enriched Gene Ontology (GO) terms in the categories of biological process (BP), cellular component (CC), and molecular function (MF). (**B**) The top 10 enriched Kyoto Encyclopedia of Genes and Genomes (KEGG) pathways associated with the potential anti-oxidative stress mechanism of FLYZ.

To elucidate the potential mechanisms by which FLYZ counteracts oxidative stress, GO and KEGG pathway enrichment analyses were performed on the 88 common targets. GO enrichment analysis yielded 595 significant terms, including 426 biological processes (BP), 58 cellular components (CC), and 111 molecular functions (MF). The top 10 enriched terms in each category, ranked by *p*-value, are presented in Figure 3A. Notably, a dot in darker red in the graphical representation corresponds to lower P, signifying a more pronounced enrichment. Based on the above principle, the dots that represent the greatest enrichment among BP, CC and MF are response to xenobiotic stimulus (GO:0009410), plasma membrane (GO:0005886), and enzyme binding (GO:0019899), respectively. As for BP, the anti-oxidative stress effects of FLYZ are mainly due to response to xenobiotic stimulus (GO:0009410), adenylate cyclase-activating adrenergic receptor signaling pathway (GO:0071880), response to hypoxia (GO:0001666), negative regulation of apoptotic process (GO:0043066), and positive regulation of apoptotic process (GO:0043065). As reported, the coordinated transcriptional regulation of human UGT1As by aryl hydrocarbon receptor (AhR) and Nrf2, involving xenobiotic and antioxidant response elements, reveals the anti-oxidative stress effect of the response to the xenobiotic stimulus pathway [29]. In addition, response to hypoxia can trigger oxidative stress, among which hypoxia-inducible factor 1α (HIF-1α) serves as a master transcription factor [30]. In terms of CC, FLYZ mainly influences plasma membrane (GO:0005886), postsynaptic membrane (GO:0045211), presynaptic membrane (GO:0042734), synapse (GO:0045202), and neuron projection (GO:0043005). Studies confirm that plasma membrane [31] and postsynaptic membrane [32] are related to the regulation of oxidative stress, demonstrating the anti-oxidative stress potential of FLYZ. For MF, the targets mostly participate in enzyme binding (GO:0019899), identical protein binding (GO:0042802), estrogen response element binding (GO:0034056), RNA polymerase II transcription factor activity, ligand-activated sequence-specific DNA binding (GO:0004879), and protease binding (GO:0002020). Previous experiments show that enzyme binding plays a vital role in anti-oxidative stress [33]. Moreover, quercetin and catechin attach to Forkhead box O3 at different sites, and thus affect the interleukin-1 receptor-associated kinase 1 alpha (IKKα)/tumor protein p53 pathway and Nrf2 pathway, confirming that identical protein binding is the key to the anti-oxidative stress process [34]. In conclusion, the findings indicate that FLYZ exerts multiple synergistic impacts on oxidative stress.

KEGG pathway analysis identified 135 significantly enriched pathways. The top 10 pathways, ranked by *p*-value, are shown in Figure 3B. The most relevant pathways for oxidative stress intervention included “Neuroactive ligand-receptor interactio (hsa04080)”, “Lipid and atherosclerosis (hsa05417)”, and “Pathways in cancer (hsa05200)”. Specifically, in the neuroactive ligand–receptor interaction pathway, target genes of the Eucommia ulmoides Oliver–Tribulus terrestris L. (EUO-TT) drug pair, such as CHRM1, interact with ferroptosis-related proteins, which may exert potential anti-oxidative stress effects [35]. Moreover, lipid and atherosclerosis are known to be closely associated with oxidative stress [36]. Collectively, the GO and KEGG analyses suggest that FLYZ may alleviate oxidative stress through a multi-target, multi-pathway mode of action, involving key biological processes, cellular localizations, and molecular interactions within several critical signaling pathways.

#### 3.1.5. Analysis of Predicted Compound–Target Interactions via Molecular Docking

**Figure 4:**
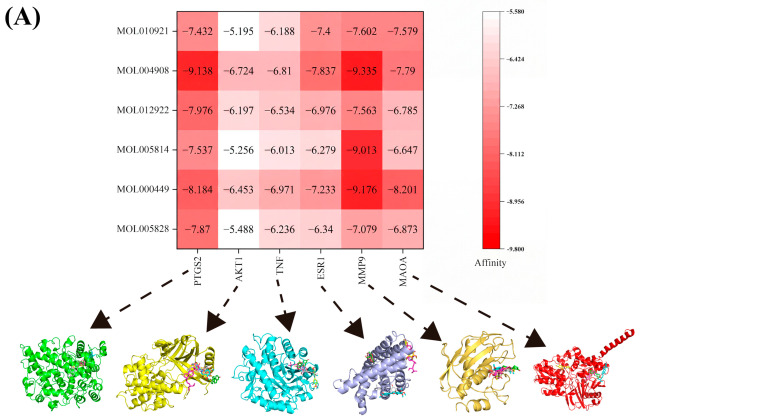

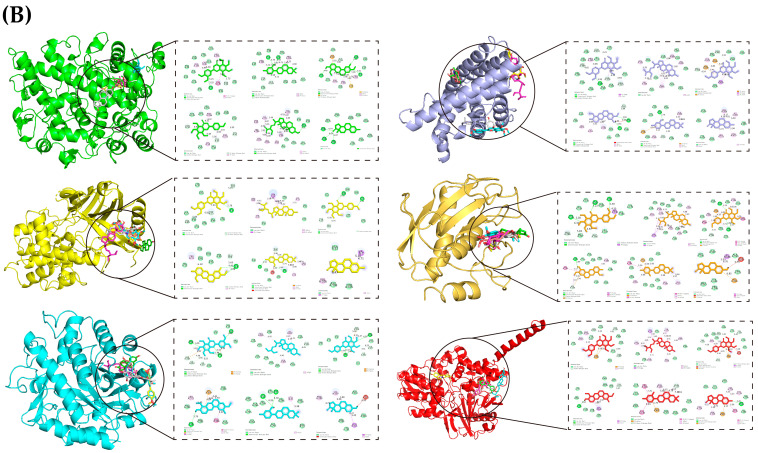
Molecular docking validation of interactions between key FLYZ compounds and hub targets. (**A**) Heatmap of the predicted binding energies for the docked complexes. (**B**) Representative three-dimensional views of the predicted binding conformations and interaction modes between the active compounds and their corresponding target proteins.

Molecular docking was performed to evaluate the potential for interaction between key FLYZ compounds and the identified hub targets [37]. The predicted binding modes and interaction sites are illustrated in Figure 4B, revealing a range of potential interactions including hydrogen bonds, van der Waals forces, and π-related interactions. The calculated binding energies for all docked complexes are summarized in Figure 4A. All compound–target pairs showed binding energies lower than −5.0 kcal/mol (PTGS2: −9.1 to −7.4; AKT1: −6.7 to −5.2; TNF: −7.0 to −6.0; ESR1: −7.8 to −6.3; MMP9: −9.3 to −7.1; MAOA: −8.2 to −6.6 kcal/mol), indicating favorable predicted binding affinities in silico [38]. These computational results suggest that the hub targets may plausibly engage with the bioactive compounds in FLYZ. It is important to note that docking provides a predictive model of interaction; these findings highlight potential mechanisms that warrant further experimental validation to confirm their biological relevance to the formula’s antioxidant activity.

### 3.2. Optimization and Validation of the FLYZ Formulation for Antioxidant Activity

#### 3.2.1. Single-Factor Experiment

**Figure 5:**
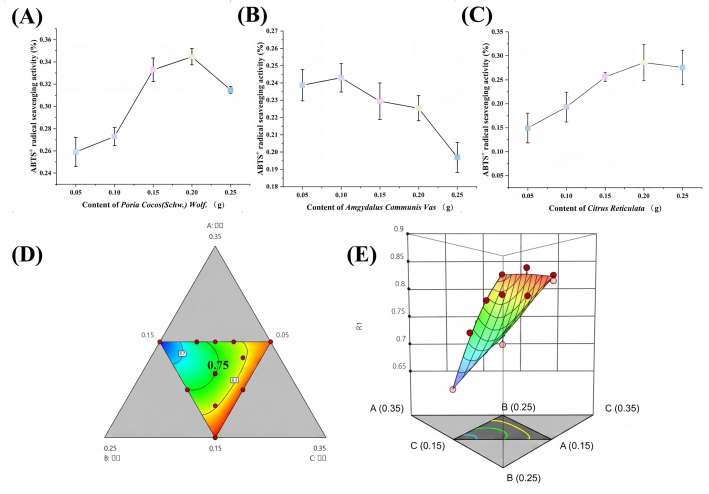
Optimization of the FLYZ formulation using single-factor experiments and response surface methodology (RSM). (**A**–**C**) Effects of individual components on ABTS^+^ scavenging activity: (**A**) *Poria cocos (Schw.) Wolf* (**A**), (**B**) *Amygdalus communis* (**B**), and (**C**) *Citrus reticulata* (**C**). (**D**) Contour plot and (**E**) 3D response surface plot showing the combined effects of the three components on ABTS^+^ scavenging activity, as predicted by the RSM model. In (**D**,**E**), A = *Poria cocos (Schw.) Wolf,* B = *Amygdalus communis*, C = *Citrus reticulat*. The color gradient in the response surface plots (**D**,**E**) represents the magnitude of the response variable R1 (ABTS radical scavenging activity). Red/orange indicates higher antioxidant activity, while blue/green indicates lower activity. The optimal formulation region is identified by the red-colored area in the contour plot (**D**), which corresponds to the peak of the 3D surface plot (**E**).

Single-factor experiments were conducted to determine the approximate optimal ranges of *Poria cocos (Schw.) Wolf.*, *Amygdalus Communis Vas*, and *Citrus Reticulata* for maximizing ABTS^+^ scavenging activity. As shown in Figure 5A–C, the highest scavenging activity was achieved when 0.20 g of *Poria cocos (Schw.) Wolf.*, 0.10 g of *Amygdalus Communis Vas*, and 0.20 g of *Citrus Reticulata* were applied. Based on these results, the following ranges were selected for subsequent response surface methodology (RSM) optimization: 0.15–0.25 g for *Poria cocos (Schw.) Wolf.*, 0.05–0.15 g for *Amygdalus Communis Vas*, and 0.15–0.25 g for *Citrus Reticulata*. This setting was conducive to further delving into the interactive relationships among diverse factors and their aggregated influence on antioxidant activity. Consequently, a scientific rationale for precise optimization of the FLYZ formula can be achieved, and the latent application prospects and underlying mechanisms of this system within the domain of diseases related to anti-oxidative stress can be more exhaustively uncovered.

#### 3.2.2. Response Surface Methodology Optimization and Model Validation

A constrained mixture design was implemented using Design-Expert 12 software to investigate the interaction effects of the three factors (Table 1). A special cubic mixture model was fitted to the experimental data (Table 2), describing the ABTS^+^ scavenging rate (Y) as a function of the amounts of *Poria cocos (Schw.) Wolf* (A), *Amygdalus communis Vas* (B), and *Citrus reticulata* (C) (with codes defined as in Section 2.3.4): Y = 21.771(A) + 44.851(B) + 22.563(C) − 250.970(AB) − 151.529(AC) − 243.601(BC) + 1009.001(ABC), where the total mass of A, B, and C is fixed at 0.55 g. Analysis of variance (ANOVA) indicated that the model was highly significant (*p* < 0.0001). The lack-of-fit was not significant (*p* = 0.5838), and the high coefficients of determination (R^2^ = 0.9695, adjusted R^2^ = 0.9492) confirmed the model’s goodness-of-fit and predictive capability. Significant interaction terms (AB, AC, BC, ABC; *p* < 0.05) demonstrated complex interplay among the three herbs in influencing antioxidant activity. The three-dimensional response surface and two-dimensional contour plots (Figure 5D,E) visually represent these interactions, with pronounced curvature in the central regions indicating strong synergistic or antagonistic effects. The three factors—*Poria Cocos (Schw.) Wolf.*, *Amygdalus Communis Vas*, and *Citrus Reticulata*—showed differential and distinctive effects on the ABTS^+^ scavenging activity, which can be traced back to their unique chemical compositions. *Poria Cocos (Schw.) Wolf.* is renowned for its rich reservoir of bioactive polysaccharides and triterpenoids [39], which are hypothesized to interact with ABTS^+^ by donating hydrogen atoms or electrons, thereby disrupting the oxidative chain reaction and quenching the radicals [40]. In addition, *Amygdalus Communis Vas* contains phenolic compounds and flavonoids [41]. The phenolic hydroxyl groups in these compounds can directly scavenge ABTS^+^ through hydrogen donation, forming more stable radical intermediates [42]. *Citrus Reticulata*, which is abundant in vitamin C and other antioxidants, plays a significant role in the overall radical scavenging process [43]. Vitamin C can regenerate oxidized antioxidants, prolonging the antioxidant defense mechanism [44].

Based on the model, the optimal formulation was predicted to be a 5:1:5 ratio of A:B:C (*Poria cocos (Schw.) Wolf*:*Amygdalus communis Vas*:*Citrus reticulata*). The RSM model thus provided a directed candidate for experimental testing. Experimental verification of this ratio yielded an average ABTS^+^ scavenging rate of 83.13%. This experimentally confirmed value, which is in reasonable agreement with the model-predicted value of 93.7%, validates the utility of the RSM approach in efficiently guiding the formulation optimization process.

#### 3.2.3. Assessment of Antioxidant Synergism Using the Chou–Talalay Method

**Figure 6:**
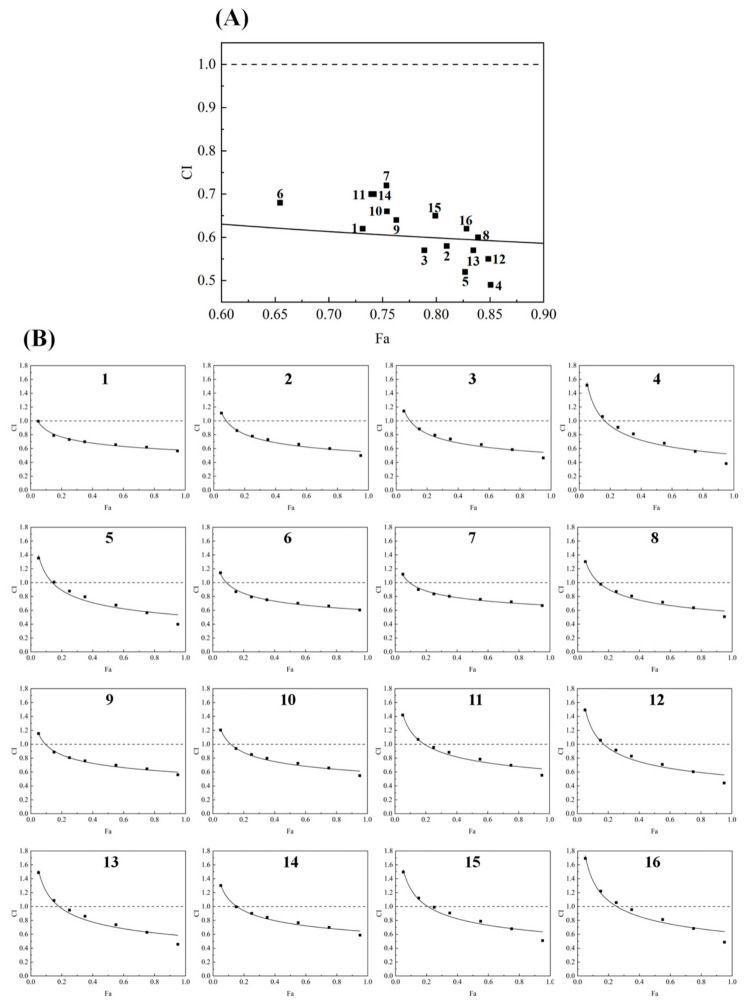
Analysis of synergistic antioxidant effects among FLYZ components. (**A**) Combination index (CI) values and (**B**) Fa-CI plots for different FLYZ formulations based on ABTS^+^ scavenging activities. The serial numbers (1, 2, 3, …) correspond to the experimental groups listed in Table 1.

The synergistic interaction of the three herbs in the FLYZ formulation was quantitatively evaluated using the combination index (CI) method [20], where CI < 1 indicates synergy, CI = 1 additivity, and CI > 1 antagonism. As shown in Figure 6A, all tested FLYZ formulations yielded CI values below 1, demonstrating a clear synergistic antioxidant effect among *Poria cocos (Schw.) Wolf.*, *Amygdalus communis*, and *Citrus reticulata*. This synergy was maintained across a range of effect levels, as illustrated in the CI-vs-Fa plot (Figure 6B). Notably, Formulation 4—which corresponds to the RSM-optimized ratio of 5:1:5—exhibited the lowest CI (strongest synergy) alongside the highest ABTS^+^ scavenging activity. These results confirm that the combined antioxidant effect surpasses the sum of the individual contributions.

It is important to note that the CI is a robust, quantitative indicator of synergy at the phenomenological level but does not disclose the underlying chemical or biochemical mechanisms. The observed synergy may be tentatively attributed to complementary antioxidant pathways and compound interactions among the constituents—for instance, through enhanced electron donation, radical stabilization, or provision of additional antioxidant moieties, as suggested in the literature [45,46,47]. Therefore, while the CI analysis establishes a clear synergistic interaction in ABTS^+^ scavenging, the precise molecular basis of this synergy remains to be elucidated and warrants dedicated mechanistic investigation in future studies. The present findings affirm that assessing synergistic effects is valuable for formulation optimization, and the optimized FLYZ ratio (5:1:5) provides a promising basis for further functional-food development.

#### 3.2.4. Cytoprotective and Antioxidant Effects of FLYZ in BNL CL.2 Cells

**Figure 7:**
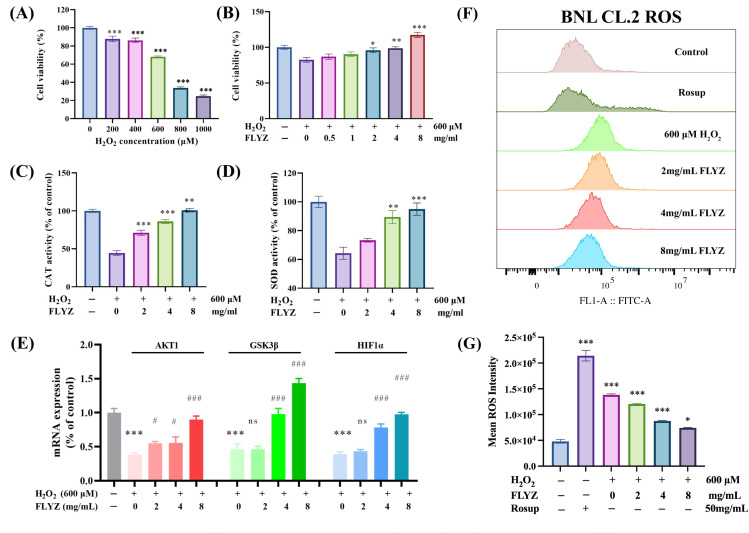
Protective effects of FLYZ against H_2_O_2_-induced oxidative damage in BNL CL.2 cells. (**A**) Cell viability after treatment with different concentrations of H_2_O_2_ (0–1000 μM). (**B**) Protective effect of FLYZ (0–8 mg/mL) on cell viability in the presence of 600 μM H_2_O_2_. (**C**) Catalase (CAT) and (**D**) superoxide dismutase (SOD) activities in cells treated with H_2_O_2_ and/or FLYZ. (**E**) Representative flow cytometry histograms and (**F**) quantitative analysis of intracellular ROS levels detected by DCFH-DA fluorescence. Data are presented as mean ± SD (*n* = 3). ns: no statistically significant difference, * *p* < 0.05, ** *p* < 0.01, *** *p* < 0.001 vs. H_2_O_2_-treated group. (**G**) Relative mRNA expression levels of AKT1, GSK3β, and HIF-1α. * *p* < 0.05, *** *p* < 0.001 vs. untreated control; ^#^ *p* < 0.05, ^###^ *p* < 0.001 vs. H_2_O_2_-treated group.

To evaluate the fundamental antioxidant and cytoprotective potential of FLYZ, we employed the non-transformed murine hepatic cell line BNL CL.2. This cell model provides a relevant system for assessing oxidative stress responses, as the liver is a central organ in xenobiotic metabolism and antioxidant defense, and the use of a non-cancerous line minimizes confounding effects from oncogenic alterations. A cellular oxidative stress model was established by treating BNL CL.2 cells with H_2_O_2_. A concentration of 600 μM H_2_O_2_, which reduced cell viability to approximately 68.3% (Figure 7A), was selected for subsequent experiments. Pretreatment with FLYZ (2–8 mg/mL) significantly attenuated H_2_O_2_-induced cytotoxicity and increased cell viability in a dose-dependent manner (Figure 7B), indicating a potent cytoprotective effect. The antioxidant capacity of FLYZ was further assessed by measuring the activities of key antioxidant enzymes. H_2_O_2_ exposure suppressed catalase (CAT) activity by 55.6% compared to the control, while FLYZ co-treatment restored CAT activity in a dose-dependent manner (Figure 7C). Similarly, FLYZ reversed the H_2_O_2_-induced decrease in superoxide dismutase (SOD) activity (Figure 7D), confirming its role in enhancing cellular antioxidant defenses. To directly evaluate intracellular oxidative stress, ROS levels were measured by flow cytometry using the DCFH-DA probe. H_2_O_2_ treatment markedly increased ROS generation, whereas FLYZ pretreatment (2, 4, and 8 mg/mL) significantly reduced ROS levels in a concentration-dependent manner (Figure 7E,F), providing direct functional evidence of its antioxidative action. To explore the potential mechanism, the expression of genes within the AKT1/GSK3β/HIF-1α pathway—identified as potential targets via network pharmacology and molecular docking—was examined by qRT-PCR. H_2_O_2_ significantly downregulated the mRNA levels of AKT1, GSK3β, and HIF-1α, whereas FLYZ treatment reversed these changes in a concentration-dependent manner (Figure 7G). Together, the restoration of antioxidant enzyme activities, reduction in intracellular ROS, and modulation of pathway-related gene expression suggest that the protective effect of FLYZ may be associated with the AKT1/GSK3β/HIF-1α axis. It is important to note that the present cellular study was designed to validate the intrinsic antioxidant bioactivity of FLYZ. While the BNL CL.2 model is appropriate for this purpose, the findings are limited to a single murine hepatic cell system. Future studies employing human-derived cell models or in vivo validation will be valuable to further establish the physiological relevance and translational potential of FLYZ.

### 3.3. Functional Performance and Nutritional Evaluation of 3D-Printed Dysphagia-Level Foods

#### 3.3.1. Assessment of Pre-Printing Gel Properties

**Figure 8:**
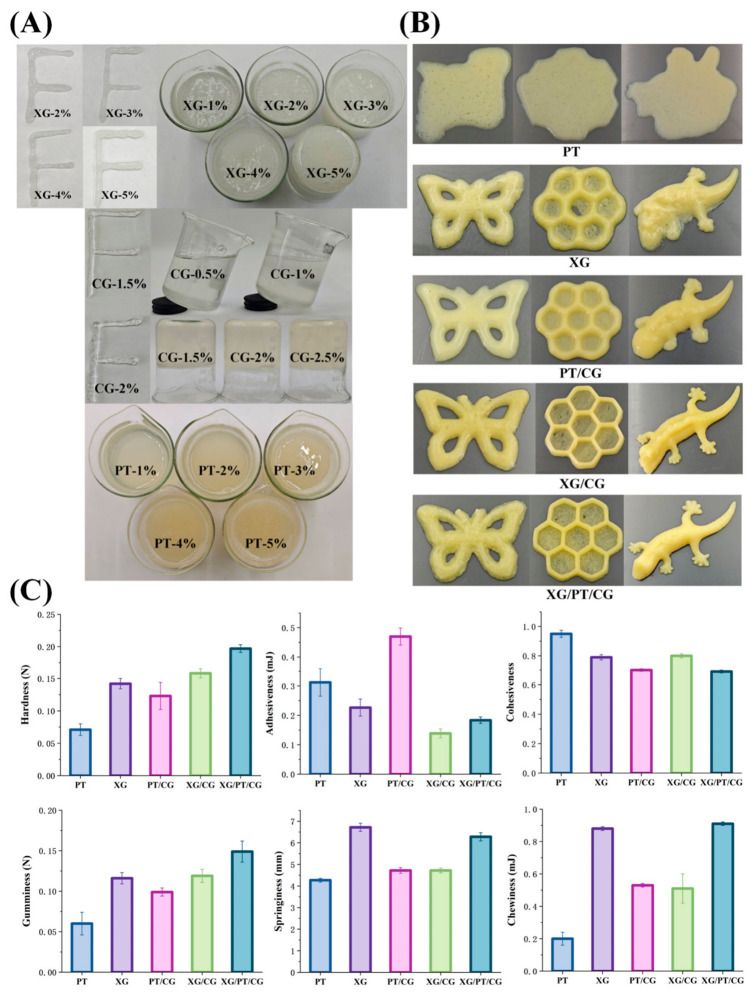
Textural and visual properties of the 3D-printed gels. (**A**) Visual appearance of the different gel formulations. (**B**) Images of the 3D-printed gel structures. (**C**) Texture profile analysis (TPA) parameters of the printed gels.

To evaluate the printability of various colloid formulations, three types of colloids were investigated under simulated extrusion conditions relevant to 3D printing (Figure 8A). Formulations containing 1 g/100 g XG exhibited poor shape retention post-extrusion. At 2 g/100 g, the printed lines showed limited self-supporting capacity, while 3 g/100 g XG formulations yielded uniform and moderately stable extrudates. Conversely, higher concentrations (4–5 g/100 g) remained in an aggregated state, suggesting incomplete hydration or dissolution. In the case of CG, concentrations of 0.5 and 1 g/100 g were insufficient to maintain structure after extrusion. A concentration of 1.5 g/100 g CG enabled uniform extrusion with moderate stability, while 2 g/100 g formulations exhibited irregularities due to increased viscosity and heterogeneity. At 2.5 g/100 g, excessive gelling likely disrupted fluidity, resulting in poor extrudability. This behavior is attributable to the thermo-reversible gelation mechanism of CG, where higher concentrations enhance network formation through ionic crosslinking in junction zones. PT-based inks displayed a fluid-like consistency across all concentrations tested. Inks containing 1–2 g/100 g PT were fully liquefied, whereas those at 4–5 g/100 g were partially aggregated, likely due to delayed hydration. Overall, 3 g/100 g XG, 1.5 g/100 g CG, and 3 g/100 g PT were identified as optimal for subsequent printing trials, with 1% oil incorporated to simulate real food matrix conditions.

#### 3.3.2. Texture Profile Analysis

TPA was used to assess key textural attributes of the formulations, including hardness, adhesiveness, cohesiveness, gumminess, springiness, and chewiness—parameters closely linked to sensory perception and consumer acceptance [48,49]. Hardness, determined by the peak force in the first compression cycle, ranged from 0.071 to 0.197 N (Figure 7C). Inks with lower hardness lacked structural integrity during printing, indicating insufficient self-support. The XG/PT/CG formulation demonstrated the highest hardness (0.197 N), followed by XG/CG (0.158 N) and XG alone (0.142 N). These results suggest that multi-colloid systems form more robust gel networks through synergistic interactions, such as hydrogen bonding and entanglement, improving structural resilience [50]. Adhesiveness, representing the negative area during probe withdrawal, varied from 0.139 to 0.470 mJ. Suitable adhesiveness aids in the release of taste and flavor attributes [51]. However, excessively adhesive inks may stick to packaging or oral surfaces, affecting usability and palatability [52]. The PT/CG combination showed the highest adhesiveness (0.470 ± 0.029 mJ), whereas XG/CG (0.139 ± 0.015 mJ) and XG/PT/CG (0.184 ± 0.011 mJ) were significantly lower, indicating more favorable textural profiles for dysphagia diets. Cohesiveness—calculated as the ratio of the positive force area during the second compression to that of the first—reflects the integrity of a food’s internal bonding and its ability to deform before fracturing. The cohesiveness of different formulations ranged from 0.69 to 0.95 g·s. Notably, the XG/PT/CG ink exhibited an ideal cohesiveness, indicating a strong internal network that resists deformation under compression. This phenomenon is likely due to enhanced crosslinking and compatibility among the three colloids. Gumminess, calculated as the product of hardness and cohesiveness, reflects the energy required to disintegrate food for safe swallowing. Springiness, measuring the capacity of samples to regain their original height post-compression, influences the perceived rubberiness of inks during oral processing [53]. Formulations with high springiness exhibited limited fragmentation into large pieces upon initial compression, whereas low-springiness counterparts disintegrated into numerous small fragments. This pattern aligned with chewiness across all ink formulations, suggesting a unified mechanical response during mastication [54]. Chewiness quantifies the mechanical energy needed to masticate solid foods until a swallowable consistency is achieved. The observed disparities in mastication behavior among ink formulations correlated with springiness variations, potentially caused by colloid-water interactions and molecular structural arrangements within the inks. These findings highlight the potential of custom-designed colloidal blends to achieve target textures suited to individuals with swallowing difficulties. Inks incorporating XG, PT, and CG showed suitable texture properties, consistent with the findings of Guan et al. [55].

#### 3.3.3. Evaluation of 3D Printing Behaviors

The esthetic appeal of dysphagia-friendly foods is crucial for enhancing patient appetite and intake. To assess printing performance, three geometries of increasing complexity were used. Printing parameters (nozzle diameter, speed, and temperature) were kept constant to isolate formulation effects (Figure 7B). Single-component inks (PT or XG) exhibited significant deformation due to insufficient mechanical strength. When CG was introduced, shape retention improved but issues like uneven extrusion and strand breakage remained. In contrast, the XG/PT/CG composite demonstrated excellent form stability and surface smoothness. The better printability can be ascribed to the improved mechanical strength and self-supporting ability of the ink achieved by colloidal blending. Compared to traditional pureed or mashed foods, the refined surface and consistent structure of 3D-printed XG/PT/CG samples offer a visually appealing alternative that may stimulate appetite and enhance the mealtime experience for individuals with dysphagia.

#### 3.3.4. IDDSI Framework Compliance Testing

Dysphagia profoundly impacts dietary intake, nutritional well-being, and the overall quality of life for elderly populations and those with swallowing difficulties. To address this, industry standards now favor the use of texture-modified foods—ranging from thickened liquids to soft or semi-solid meals—formulated to match each person’s oral processing abilities and nutritional needs. A key metric in assessing these modified foods is the structure degree, which encompasses both textural (hardness, cohesiveness, adhesiveness) and particulate (particle size) characteristics of the 3D-printed food. The IDDSI classifies foods into eight levels to guide safe selection. It provides simple tests (spoon tilt for cohesiveness, adhesiveness, and hardness, fork drip and fork pressure for particle size and hardness) that yield indirect but clinically useful information on these properties.

Based on IDDSI, spoon tilt tests evaluated food cohesiveness and adhesiveness critical for dysphagia safety [56]. Overly cohesive formulations risk pharyngeal retention, while low-cohesion materials may disperse unpredictably—both elevating aspiration hazards [57,58]. Optimal texture-modified diets should maintain shape on the spoon, slide off intact without residue, and minimize stickiness to prevent mucosal adhesion during swallowing. Single-colloid samples exhibited excessive adhesiveness, adhering to spoons and leaving substantial residues. The addition of CG slightly reduced stickiness, while PT/CG blends remained unsuitable due to increased transit resistance and required excessive tongue force for pharyngeal movement. In contrast, the XG/PT/CG ink retained form on spoons, slid off cleanly without deformation or residue, and lacked liquid-solid separation or lumps (Table S2), which aligns with level 4 pureed/extremely thick foods—non-pourable, shape-retaining, and swallowable without chewing—mitigating risks of sticking or aspiration by facilitating smooth oral transit. The formulation’s balanced cohesiveness and low adhesiveness demonstrate compliance with clinical standards for safe dysphagia foods.

The fork drip test assesses ink flow behavior using fork tines, where PT and PT/CG formulations exhibited gradual flow through the prongs, forming short tails beneath the fork. In contrast, other inks remained piled on the fork without significant flow. The fork pressure test evaluates food hardness and fragmentation under utensil pressure, conducted on 15 × 15 × 15 mm^3^ 3D-printed cubes—dimensions aligned with adult choking safety standards. Using a standard fork (4 mm inter-tine gap, matching the 2–4 mm safe pellet size for swallowing), XG/CG and XG/PT/CG samples were easily mashed with minimal thumb pressure (no nail blanching), indicating tongue-compressible textures [59]. Their unobstructed passage through the 4 mm slots confirmed particle sizes within safe ranges [60]. These results align with level 4 pureed/extremely thick foods within the IDDSI framework, supporting the classification of XG/CG and XG/PT/CG formulations into this safety category. It is important to note that this study did not perform instrumental rheological characterization (e.g., viscosity, shear-thinning behavior, yield stress, or viscoelastic moduli) of the 3D printing inks. Such measurements would be valuable for a more mechanistic understanding of extrudability and shape retention, and they represent an important direction for future research. The present conclusions on printability are based on TPA and visual observations, which are sufficient for screening formulations for dysphagia-friendly texture and 3D printing feasibility.

#### 3.3.5. Antioxidant Activity of 3D-Printed Gels

**Figure 9:**
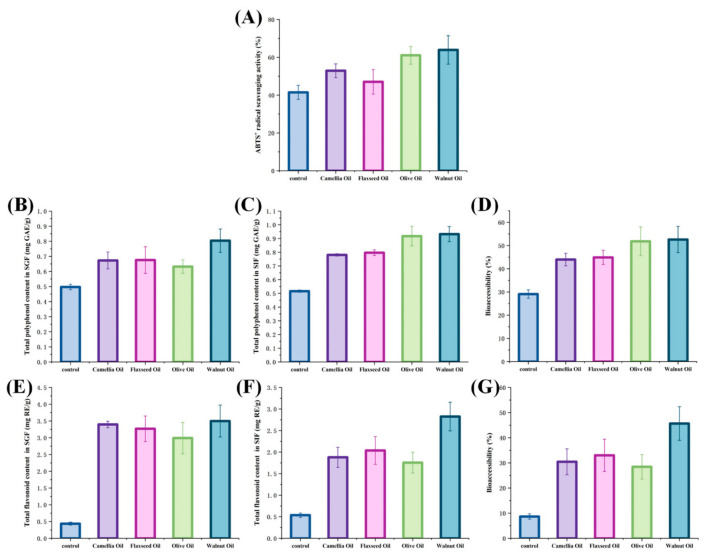
Antioxidant properties and bioaccessibility of phenolic compounds in 3D-printed gels after in vitro digestion. (**A**) ABTS^+^ radical scavenging activities of gels with different oil formulations. Total phenolic content in the (**B**) simulated intestinal fluid (SIF) and (**C**) simulated gastric fluid (SGF) fractions. (**D**) Bioaccessibility of phenolic compounds after intestinal digestion. Total flavonoid content in the (**E**) SIF and (**F**) SGF fractions. (**G**) Bioaccessibility of flavonoids after intestinal digestion.

ABTS^+^ radical scavenging assays were conducted in vitro to evaluate the antioxidant capacity of various 3D-printed gels. As shown in Figure 9A, all FLYZ-based formulations demonstrated strong ABTS^+^ scavenging activity. Notably, incorporating walnut oil into the XG/PT/CG gel matrix boosted the scavenging rate from 41.47% in the control to 63.92%, reflecting both the intrinsic antioxidant capacity of walnut oil and the benefits of its complexation within the gel network. This 22.45% enhancement parallels findings by Mohammadi et al., confirming that walnut oil–based gels markedly improve ABTS^+^ radical scavenging capacity. These results underscore the potent antioxidant potential of FLYZ-loaded 3D-printed gels and support their promise as potent antioxidant agents [61].

#### 3.3.6. Retention of Total Phenolic and Flavonoid Contents After In Vitro Digestion

For bioactive substances to function effectively in the body, absorption by the small intestine is crucial. Yet, the acidic gastric environment poses a major threat, often causing degradation during digestion. This study employed an in vitro digestion model to investigate how gels affect the TPC and TFC of FLYZ. The control showed the lowest TPC (0.516 mg GAE/g) and TFC (0.537 mg RE/g), likely due to direct degradation by SGF and SIF (Figure 9B,C,E,F). In contrast, colloid-crosslinked samples under the same digestion conditions showed TPC ranging from 0.781 to 0.932 mg GAE/g and TFC from 1.758 to 2.826 mg RE/g. Significantly, 3D-printed gels enhanced the TPC and TFC of FLYZ, demonstrating the protective role of colloid crosslinking against polyphenol and flavonoid degradation. Colloids act as physical barriers, shielding these bioactive compounds from environmental stress and promoting their stability post-encapsulation. Additionally, gels with different oil types released bioactive substances differently in SIF. The degradation of gels was closely related to their chemical structures and gel network properties. During simulated digestion, gels containing various oils offered distinct protective effects. Notably, the XG/PT/CG gel with walnut oil showed the best protective effects, maintaining a TPC of 0.932 mg GAE/g and a TFC of 2.826 mg RE/g after digestion. This finding aligns with previous research, suggesting that the walnut oil-based XG/PT/CG gel can regulate bioactive substance release in gastrointestinal conditions [62]. The stable gel structure likely stems from its intact bond networks in acidic environments, whereas unencapsulated samples degraded faster due to compromised structural integrity [63]. The present study provides further evidence for the integrated functional benefits of the FLYZ formulation and demonstrates the applicability and advantages of 3D printing in fabricating such functional herbal products.

#### 3.3.7. Analysis of the Bioaccessible Fraction

Bioaccessibility describes the fraction of bioactive compounds liberated from a food matrix during digestion and made available for uptake into the bloodstream or cells [64]. As shown in Figure 9D,G, unencapsulated FLYZ showed the lowest bioaccessibility—polyphenols at 29.08% and flavonoids at 8.68%. It is well-known that colloidal carriers can shield sensitive ingredients and enhance their release profiles [65,66]. Accordingly, embedding FLYZ in 3D-printed gels stabilized by XG, PT, and CG markedly improved its bioaccessibility. In particular, the walnut oil–fortified XG/PT/CG gels elevated polyphenol and flavonoid bioaccessibility to 52.61% and 45.69%, respectively—likely due to a tighter intermolecular network that slows degradation during digestion. Compared with conventional molding, 3D printing allows precise control over processing conditions, more uniform encapsulation of FLYZ, and better protection from oxidative or photolytic damage during fabrication and storage. Furthermore, the layered architecture of 3D printing gels may regulate the release rate of polyphenols and flavonoids, promoting their gradual liberation throughout digestion. In summary, the synergistic formulation of XG, PT, CG, and walnut oil in a 3D-printing matrix effectively preserves and enhances the bioaccessibility of key antioxidants in FLYZ, thereby increasing their potential uptake into the human body [67].

## 4. Conclusions

This study demonstrates the potential of Fu Ling Yin Zi (FLYZ) as a basis for a 3D-printable functional food. An optimized formulation (Poria cocos: Amygdalus communis: Citrus reticulata = 5:1:5) exhibited high antioxidant activity, and the Chou–Talalay analysis confirmed a synergistic interaction among its components. Predictive network pharmacology and docking analyses suggested potential interactions with targets like AKT1, and subsequent cellular assays showed that FLYZ enhanced antioxidant enzyme activities, reduced intracellular ROS, and modulated the expression of genes associated with the AKT1/GSK3β/HIF-1α axis, supporting a potential role of this pathway. A functional walnut oil–based 3D-printing hydrogel was successfully developed, with its texture classified as IDDSI level 4 (pureed/extremely thick) using standardized fork and spoon tests. The printed gel also protected the bioactive compounds during simulated digestion, improving their bioaccessibility. Collectively, this work integrates bioactive formulation optimization with advanced food manufacturing, advancing the development of texture-modified, antioxidant-functional foods for personalized nutrition, with future clinical studies warranted to translate these findings into practical dietary applications.

## Figures and Tables

**Table 1:** Experimental design and results of the response surface methodology for optimizing the ABTS^+^ scavenging activity.

Run NO.	A/g	B/g	C/g	ABTS^+^ Radical Scavenging Activity (%)
1	0.20	0.15	0.20	73.15
2	0.18	0.13	0.24	80.98
3	0.25	0.08	0.22	78.89
4	0.20	0.10	0.25	85.06
5	0.25	0.05	0.25	82.68
6	0.25	0.15	0.15	65.46
7	0.20	0.15	0.20	75.36
8	0.15	0.15	0.25	83.89
9	0.20	0.15	0.20	76.28
10	0.25	0.10	0.20	75.41
11	0.22	0.12	0.21	73.95
12	0.15	0.15	0.25	84.85
13	0.20	0.10	0.25	83.45
14	0.25	0.12	0.18	74.18
15	0.23	0.08	0.24	79.91
16	0.20	0.10	0.25	82.82

note: A: *Poria cocos (Schw.) Wolf* (g); B: *Amygdalus communis Vas* (g); C: *Citrus reticulata* (g). Total mass of A + B + C = 0.55 g. Repeating decimals were approximated and rounded as necessary for practical computation.

**Table 2:** ANOVA of the fitted regression model for significance evaluation.

Source	Sum of Squares	df	Mean Square	F-Value	*p*-Value	Significance
Model	0.0430	6	0.0072	47.76	<0.0001	****
Linear Mixture	0.0411	2	0.0206	137.00	<0.0001	****
AB	0.0015	1	0.0015	9.84	0.0120	*
AC	0.0015	1	0.0015	10.17	0.0110	*
BC	0.0013	1	0.0013	8.58	0.0168	*
ABC	0.0014	1	0.0014	9.53	0.0130	*
Residual	0.0014	9	0.0002			
Lack of Fit	0.0005	4	0.0001	0.78	0.5838	ns
Pure Error	0.0008	5	0.0002			
Cor Total	0.0444	15				
R^2^	0.9695					
Adjusted R^2^	0.9492					

note: A: *Poria cocos (Schw.) Wolf*; B: *Amygdalus communis Vas*; C: *Citrus reticulata*. The model is a special cubic mixture model without intercept. ANOVA based on a mixture model (special cubic) for constrained components. The ‘Linear Mixture’ row tests the significance of the linear blending terms. Lack-of-fit test supports model adequacy ****, *p* < 0.0001; *, *p* < 0.05; ns, non-significance.

## Data Availability

The original contributions presented in this study are included in the article/Supplementary Material. Further inquiries can be directed to the corresponding authors.

## Supplementary Materials

The following supporting information can be downloaded at https://www.mdpi.com/article/10.3390/foods15122206/s1, Table S1: Key bioactive compounds with the highest degree of connectivity in the FLYZ network pharmacology analysis; Table S2: International Dysphagia Diet Standardization (IDDSI) tests on 3D printing inks with modified texture for patients with dysphagia.
