# Supplementary material for: Decoding the Multi-Component Synergy of Fu Ling Yin Zi for Anti-Oxidative Stress Applications: Formulation Optimization, Molecular Docking, Cell-Based Validation, and 3D-Printed Dysphagia-Friendly Diets
Source: Foods. 2026 Jun 18;15(12):2206. doi: 10.3390/foods15122206 (PMC13297913; doi:10.3390/foods15122206)
Supplement: Supplementary file 1 [file foods-15-02206-s001.zip › foods-4337497-supplementary.pdf]

(Supplementary Information)

Research Article

Table S1. Key bioactive compounds with the highest degree of connectivity in the FLYZ network pharmacology analysis

**Compound name**	MOL ID	**degree**
nobiletin	MOL005828	**29**
stigmasterol	MOL000449	**26**
tangeretin	MOL005814	**24**
l-SPD	MOL012922	**24**
glabridin	MOL004908	**21**
estrone	MOL010921	**21**

Table S2. International Dysphagia Diet Standardization (IDDSI) tests on 3D printing inks with modified texture for patients with dysphagia.

Samples	Spoon tilt test	Fork drip test	Fork pressure test	Conclusion
PT	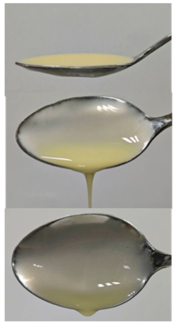 Samples exhibited excessive adhesiveness, adhering to spoons and leaving substantial residues.	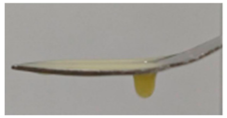 Sample exhibited gradual flow through the prongs, forming short tails beneath the fork.	/	Not suitable for dysphagia diets
XG	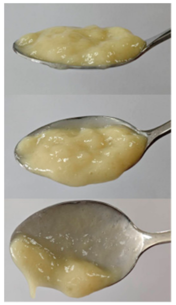 Samples exhibited excessive adhesiveness, adhering to spoons and leaving substantial residues.	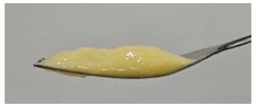 Sample remained piled on the fork without significant flow.	/	Not suitable for dysphagia diets

PT/CG	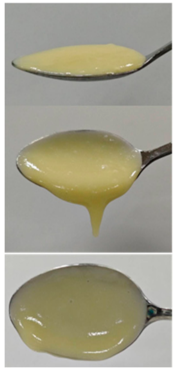	Samples exhibited excessive adhesiveness, adhering to spoons and leaving substantial residues.	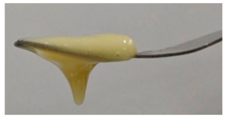	Sample exhibited gradual flow through the prongs, forming short tails beneath the fork.	/		Not suitable for dysphagia diets
XG/CG	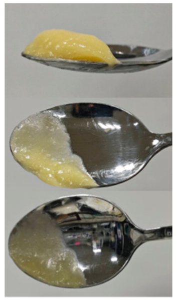	Samples retained form on spoons, slid off cleanly without deformation or residue, and lacked liquid-solid separation or lumps.	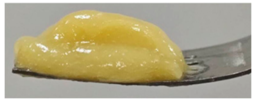	Sample remained piled on the fork without significant flow.	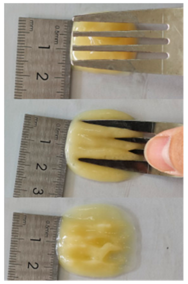	Samples were easily mashed with minimal thumb pressure (no nail blanching).	level 4 - puree/extremely thick foods
XG/PT/CG	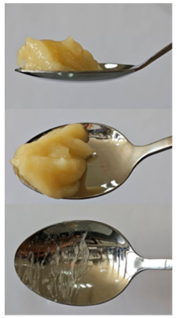	Samples retained form on spoons, slid off cleanly without deformation or residue, and lacked liquid-solid separation or lumps.	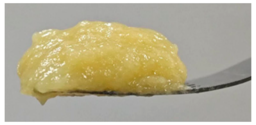	Sample remained piled on the fork without significant flow.	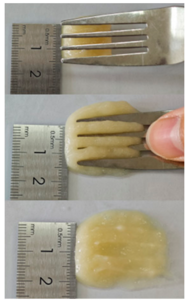	Samples were easily mashed with minimal thumb pressure (no nail blanching).	level 4 - puree/extremely thick foods
